# The Anatomical Position of Graf’s Standard Plane and Its Relationship With Pelvic Morphology: A Computed Tomography-Based Study

**DOI:** 10.7759/cureus.27424

**Published:** 2022-07-28

**Authors:** Masanori Wako, Hiroyuki Kono, Kensuke Koyama, Taro Fujimaki, Naoto Furuya, Hirotaka Haro

**Affiliations:** 1 Department of Orthopedic Surgery, University of Yamanashi, Chuo, JPN; 2 Department of Orthopedics, Yamanashi Prefectural Central Hospital, Kofu, JPN

**Keywords:** pelvic morphology, standard plane, ultrasonography, graf method, developmental dysplasia of the hip

## Abstract

Objective

The Graf method is the most widely used ultrasonographic method for evaluating developmental dysplasia of the hip (DDH), and it relies on a set standard plane. However, no previous reports have discussed the detailed anatomical location of the plane. The aim of this study was to evaluate the exact anatomical position of Graf’s standard plane in the pelvis and to ascertain the correlation between this position and pelvic morphology in children without abnormal pelvic morphology.

Methods

We retrospectively assessed the pelvic CT data of 32 children (64 hips) aged three to five years without abnormal pelvic morphology and measured the pelvic winging and acetabular anteversion and coverage. We defined the coronal plane that passed through the center of the bilateral femoral head as plane A. We determined that Graf’s standard plane could be approximated by rotating plane A until the outer wall of the ilium was parallel to the sagittal plane, and we defined this as plane A′. AA′ was defined as the angle from plane A to plane A′ on the sagittal plane. The anterior rotational angle (clockwise, viewing from the right side) was measured as the positive AA′. Moreover, we measured the pelvic rotation, acetabular anteversion, and acetabular coverage and evaluated the correlation between AA′ and these morphological parameters.

Results

The average AA′ was -8.27° and AA′ had a significant correlation with acetabular anteversion (Spearman’s ρ=0.40**, p<0.01).

Conclusions

We found that Graf's standard plane, as determined by the CT scan, tilts slightly posteriorly. This information may be useful in improving the ease of ultrasonographic examination of DDH.

## Introduction

In newborn infants, ultrasound (US) imaging of the hip joint is widely used for the early detection of developmental dysplasia of the hip (DDH). Presently, the Graf method is the most widely used standard for DDH evaluation worldwide. This method is based on the measurement of a bone angle (alpha) and a soft-tissue angle (beta) from a coronal two-dimensional (2D) US image on the standard plane [1]. Accordingly, Graf explained that the principles of the ‘standard plane’ were strongly based on: (1) the lower limb of the bony ilium in the depth of the acetabular fossa, (2) the mid-portion of the acetabular roof, and (3) the acetabular labrum [2]. Graf further stated that the lower limb of the bony ilium refers to the center of the acetabulum, that the mid-portion of the acetabular roof should be straight and parallel to the probe, and that the standard plane cannot be obtained if the probe is rotated anteriorly or posteriorly.

Despite the importance of the standard plane setting in the performance of the Graf method, to the best of our knowledge, no previous report has discussed the detailed anatomical location of the standard plane and the relationship of this position with pelvic morphology. Moreover, although it is stated that the standard plane cannot be obtained if the probe is rotated anteriorly or posteriorly, there is no report on how much the standard plane is tilted with respect to the pelvis.

It is also reported that the Graf method has low inter- and intra-observer agreement because it is highly dependent on the ability of the examiner [3-7]. If the exact location of the standard plane in the pelvis is identified, it may contribute to improving the accuracy of the Graf method.

Therefore, the present study aimed to use computed tomography (CT) data to evaluate the exact anatomical position of Graf’s standard plane in the pelvis and to ascertain the correlation between this position and pelvic morphology in children without abnormal pelvic morphology.

## Materials and methods

**Figure 1:**
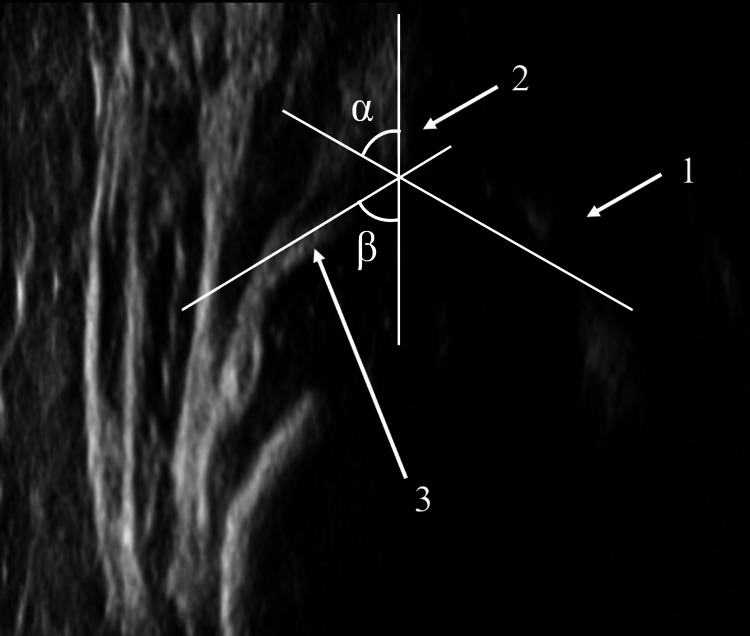
Sonographic findings of the infant hip joint Sonographic image of the infant hip on Graf’s standard plane. The alpha and beta angles of Graf's method are also shown in this figure. 1: Lower limb of the os ilium. 2: Mid-portion of the acetabular roof. 3: Acetabular labrum.

 Figure 1 shows the sonographic image of the infant hip on Graf’s standard plane.

This retrospective study was approved by the ethical committee of our university (no. 1106); the requirement for informed patient consent was waived due to the retrospective nature of the study. All experiments were performed in accordance with relevant guidelines and the regulations of our university. The investigation was based on CT images of the pelvis that were obtained for several reasons, including abdominal diseases (e.g. abdominal tumor, leukemia, and appendicitis), between 2009 and 2017 and were stored in a database. Japanese patients aged three to five years were included in the study. Patients with a history of hip-related symptoms, obvious abnormalities in skeletal development, or a history of treatment that affected bone growth were excluded. Finally, 32 eligible patients with normal pelvic morphology were retrospectively assessed. The measurements and statistical analysis were performed on the 64 hips of these 32 patients.

We reconstructed the CT data of each patient from Digital Imaging and Communications in Medicine (DICOM; NEMA (National Electrical Manufacturers Association, Rosslyn, Arlington)) files using a processing and analysis software program (SYNAPSE VINCENT, Fujifilm, Tokyo, Japan) and measured multiple parameters of pelvic morphology. To eliminate possible measurement errors, the pelvic position was digitally corrected as follows. In the coronal plane, the pelvis was horizontally aligned with a line connecting the inferior aspects of the bilateral pelvic teardrops, whereas, in the axial plane, the pelvis was vertically aligned with a line connecting the pubic symphysis and the center of the sacrum. The pelvic inclination in the sagittal plane was then aligned with a line connecting both anterior superior iliac spines (ASIS) and the pubic tubercle.

**Figure 2:**
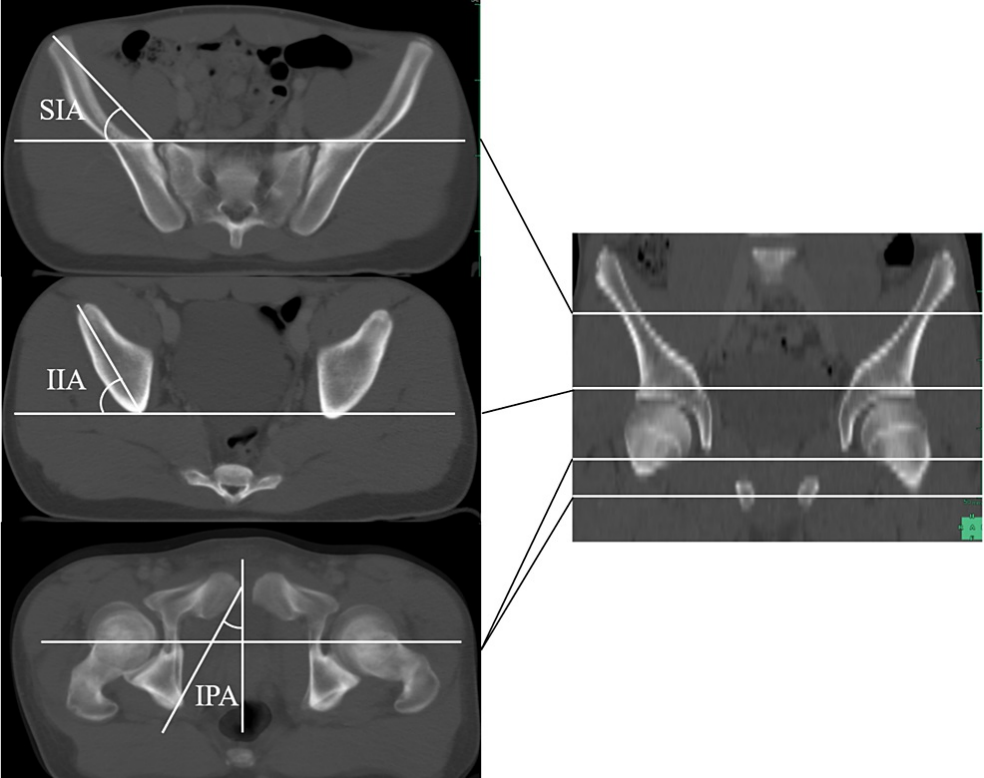
Pelvic winging Superior iliac angle (SIA), inferior iliac angle (IIA), and ischiopubic angle (IPA) as parameters indicating pelvic winging

**Figure 3:**
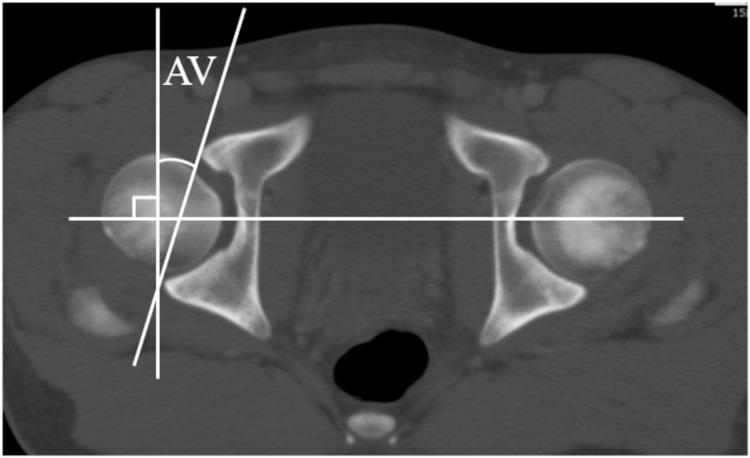
Acetabular anteversion AV is the acetabular anteversion at the level of the center of the femoral head.

**Figure 4:**
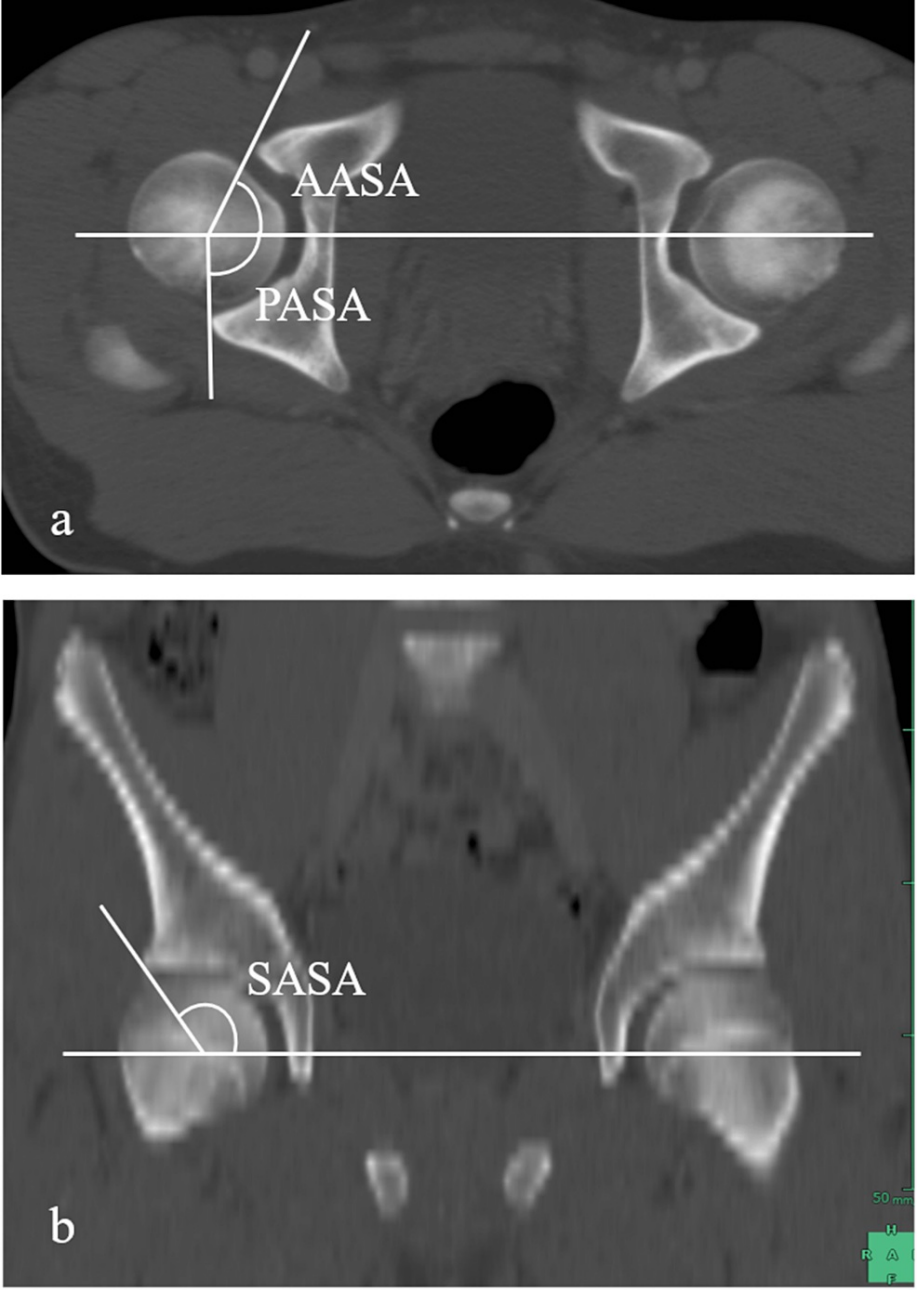
Acetabular sector angle The acetabular sector angle is formed by the intersection of a line connecting the center of the femoral head and the acetabular edge. The anterior and posterior acetabular sector angles were measured in the axial plane (a) and the superior acetabular sector angle was measured in the coronal plane (b). AASA: anterior acetabular sector angle; PASA: posterior acetabular sector angle; SASA: superior acetabular sector angle

We measured pelvic rotational alignment acetabular anteversion (AV) and acetabular coverage using the same method we reported in the past [8-9]. The following is a brief description of the specific method of measurement. As parameters of rotational alignment of the innominate bone (i.e. pelvic winging), we measured the superior iliac angle (SIA), inferior iliac angle (IIA), and ischiopubic angle (IPA), as described by Fujii et al. [10]. SIA is formed by the intersection of a line connecting the medial edge of the ASIS, the anterior margin of the sacroiliac joint, and a horizontal line on the axial plane. IIA is formed by a line connecting the anterior aspect of the anterior inferior iliac spine, the posterior aspect of the ilium, and a horizontal line on the axial plane. IPA is a projection angle formed by the intersection of a line connecting the anterosuperior edge of the pubic symphysis, the ischial spine, and a sagittal line on the axial plane, for which we superimposed the sections that passed through the ischial spine and pubic symphysis (Figure 2). AV was defined as the acetabular anteversion on the axial slice of the acetabulum corresponding to the center of the femoral head (Figure 3). We also measured the anterior acetabular sector angle (AASA), posterior acetabular sector angle (PASA), and superior acetabular sector angle (SASA) as parameters of acetabular coverage on the femoral head (Figures 4a-4b), as described by Fujii et al. [10] and Anda et al. [11]. Here, we used a horizontal line as the measurement baseline and determined the angles in the anterior, superior, and posterior directions.

**Figure 5:**
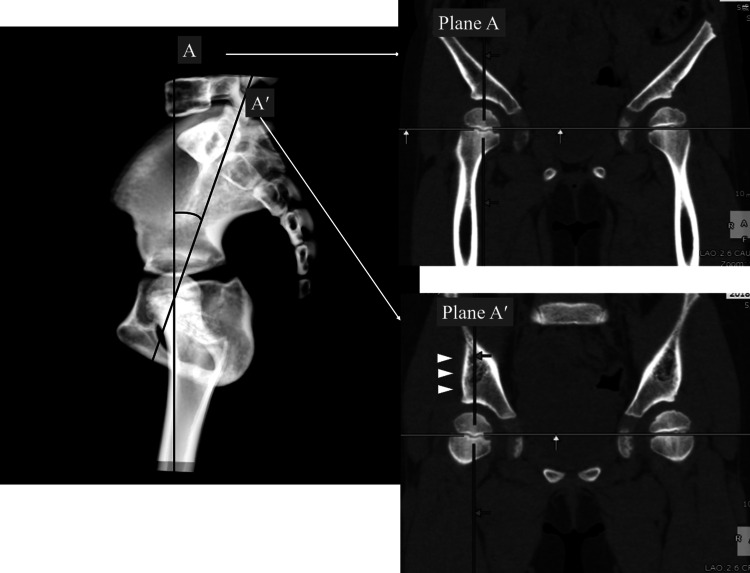
Standard plane on computed tomography The iliac contour is not parallel to the sagittal plane in plane A but is parallel to the sagittal plane in plane A′ (white allow heads). Angle AA′ in this figure is negative because plane A′ is obtained by rotating plane A clockwise, viewing from the left side.

Subsequently, we examined the detailed position of Graf’s standard plane on pelvic CT images. We initially defined the coronal plane that passed through the center of the bilateral femoral head as plane A. The inclination of the iliac outer wall proximal to the acetabulum changes as plane A is rotated on the sagittal axis passing through the center of the bilateral femoral head. As mentioned above, the true position of Graf’s standard plane is defined as a plane passing through the center of the hip joint, with an acetabular roof straight and parallel to the probe. Therefore, we determined that Graf’s standard plane could be approximated by rotating plane A until the outer wall of the ilium was parallel to the sagittal plane, and we defined this as plane A′. We then defined AA′ as the angle from plane A to plane A′ on the sagittal plane (Figure 5). The anterior rotational angle (viewing from the right side of the patient) was measured as the positive AA′.

Statistical analysis was performed using SPSS (version 25.0, IBM Corp., Armonk, NY). Spearman’s correlation coefficient was used to examine the correlations between AA′ and other parameters of pelvic morphology. A significant correlation was defined as an absolute correlation coefficient of ≥0.4 and a p-value of <0.05, whereas a significant, but weak, correlation was defined as an absolute correlation coefficient between 0.2 and 0.4 and a p-value of <0.05. To evaluate intra-observer agreement, all the measurements of 20 hips from 10 randomly selected patients were repeated by a single reader (M.W.) during two sessions separated by an interval of ≥1 month. To evaluate inter-observer agreement, a second reader (H.K.) repeated the measurements for the same 20 hips from 10 patients. The inter and intra-observer reliabilities for pelvic measurements were assessed by estimating the intraclass correlation coefficients (ICCs) along with 95% confidence intervals using the ICCs (2,1) modeling scheme.

## Results

Both intra and inter-observer correlations indicated substantial agreement (ICC >0.7) for all measurements (Table 1). Table 2 summarizes the measurements in all the patients (16 female and 16 male patients). The average AA′ was −8.27°. Table 3 presents Spearman’s correlation coefficients between AA′ and other parameters of pelvic morphology. Notably, AA′ exhibited a significant correlation with AV and demonstrated significant but weak correlations with the anterior and posterior acetabular sector angles.

**Table 1:** Reliability of each measurement (N = 20) *The values are presented as intra-class correlation coefficients, with 95% confidence intervals in parentheses. SIA: superior iliac angle, IIA: inferior iliac angle, IPA: ischiopubic angle, AV: acetabular anteversion, AASA: anterior acetabular sector angle, PASA: posterior acetabular sector angle, SASA: superior acetabular sector angle, AA´: angle between planes A and A´

Variable	Inter-observer*	Intra-observer*
SIA	0.927 (0.826, 0.970)	0.941 (0.861, 0.976)
IIA	0.890 (0.510, 0.965)	0.958 (0.899, 0.983)
IPA	0.787 (0.544, 0.909)	0.717 (0.419, 0.877)
AV	0.839 (0.037, 0.958)	0.837 (0.639, 0.932)
AASA	0.920 (0.811, 0.967)	0.951 (0.883, 0.980)
PASA	0.952 (0.883, 0.981)	0.975 (0.940, 0.990)
SASA	0.900 (0.768, 0.959)	0.926 (0.825, 0.970)
AA´	0.840 (0.638, 0.934)	0.958 (0.898, 0.983)

**Table 2:** Mean values of all morphologic variables and age SIA: superior iliac angle, IIA: inferior iliac angle, IPA: ischiopubic angle, AV: acetabular anteversion, AASA: anterior acetabular sector angle, PASA: posterior acetabular sector angle, SASA: superior acetabular sector angle, AA´: angle between planes A and A´

	Mean	SD	Range
Age (yrs)	4.28	0.89	3–5
SIA (º)	39.89	6.40	27–50
IIA (º)	57.54	6.36	44–70
IPA (º)	32.17	3.02	25–39
AV (º)	12.94	3.86	2–20
AASA (º)	52.56	5.30	42–64
PASA (º)	76.55	6.28	62–87
SASA (º)	108.02	6.79	94–126
AA´ (º)	-8.27	5.81	-17–10

**Table 3:** Spearman’s correlation coefficients between AA´ and other parameters *: p <0.05; **: p <0.01 SIA: superior iliac angle, IIA: inferior iliac angle, IPA: ischiopubic angle, AV: acetabular anteversion, AASA: anterior acetabular sector angle, PASA: posterior acetabular sector angle, SASA: superior acetabular sector angle

Variable	Spearman’s correlation coefficient
Age	0.02
SIA	-0.10
IIA	-0.17
IPA	0.08
AV	-0.40**
AASA	0.36*
PASA	-0.27*
SASA	0.12

## Discussion

In this study, we set the approximate Graf’s standard plane (plane A′) on the CT image as the plane passing through the centers of both femoral heads and the outer iliac line proximal to the acetabulum, which was parallel to the sagittal plane. We then evaluated the position of this plane and its relationship with pelvic morphology on pelvic CT images in children without abnormal pelvic morphology. We could obtain plane A′ by rotating the coronal plane posteriorly by an average of 8.27°. Moreover, this rotational angle had a significantly moderate correlation with the acetabular anteversion (Spearman’s correlation coefficients: -0.40) and a weak correlation with the anterior and posterior acetabular coverage (Spearman’s correlation coefficients: 0.36 and -0.27). There have been no reports on the anatomical location of the standard plane or the coordinate of the plane in the pelvis and the relationship of this position with the morphology of the pelvis. The results of the current study suggest that the position of the standard plane reflects the acetabular direction on the axial plane.

Moreover, although the Graf method, which is based on the measurements of the alpha and beta angles, is the most commonly used for the US diagnosis of DDH, some reports have described limitations with this method. Specifically, this method is fundamentally limited by the use of a 2D image to represent a complex 3D structure by the use of manual US probes. These characteristics may be dependent on the radiologist’s experience and can result in subjective measurements of Graf’s alpha and beta angles, subsequently increasing the risk of poor inter- and intra-observer agreement [3-7]. Several researchers have attempted to compensate for these limitations using 3D US or automatically corrected US images to evaluate DDH [12-16]. For example, Hareendranathan et al. proposed a new 2D US image-processing technique in which the bony surface was semi-automatically traced and two indices were automatically calculated: a contour-based alpha angle and a new modality-independent quantitative rounding index [12]. Furthermore, de Luis-Garcia and Alberola-Lopez [13] segmented the femoral head using an energy function based on gray-level and textural information from 3D US images, whereas Quader et al. [14] proposed a computational image analysis technique that automatically identifies adequate 2D US images and extracts metrics of dysplasia and stated a slight bias towards higher Graf categories relative to the manually estimated metrics, which could potentially reduce missed early diagnoses. However, all these reports describe the derivation of an accurate Graf’s standard plane and do not state the detailed location of the plane in the pelvis. According to the results of this study, the angle between the coronal plane, which passes through the bilateral ASIS, the pubic symphysis, and the standard plane was -10° to 17° posteriorly (mean angle: 8.27°), although there were some individual differences. Based on our findings, we suggest that applying the US probe by tilting slightly posteriorly from the coronal plane is useful for reducing the difficulty of the Graf method and for minimizing measurement errors.

This study has several limitations. The first limitation is the age of the patients. Specifically, infants targeted for DDH screening may not have a pelvic morphology similar to that of children aged three to five years. However, very few patients aged <1 year had undergone a pelvic CT examination at our institution, to make meaningful assessments of pelvic morphology. Moreover, CT-based evaluations of pelvic morphology in infants are challenging and tend to be inaccurate because of the large amount of cartilage. Further, we used data from slightly older children (aged 3-5 years) to overcome this issue. There have been some reports that pelvic morphology slightly changes between zero and three years of age [17-19], but the morphological change is small. Weiner et al. reported that the acetabular anteversion remains almost the same from zero to five years of age [18], and in the reports of Li et al., it is shown that anteversion and acetabular coverage change only about 1 degree bony from zero to five years of age [19]. Huseynovr et al. estimated pelvic morphology from infant to elderly person and stated that pelvic morphology changes little until around age 10, after which it changes significantly for each sex [20]. Therefore, we believe that our patient selection in this examination of Graf’s standard plane using pelvic CT data of patients aged three to five years has some validity. However, further studies using pelvic CT or magnetic resonance images in healthy infants aged <1 year should be performed in detail to prove this limitation. Second, the differences in pelvic morphology between different races were not considered in this study. Since the difference in pelvic morphology may affect the inclination of the standard plane, further investigation is needed in this regard.

## Conclusions

In conclusion, we reported the location of Graf’s standard plane using the CT data of healthy children aged three to five years. To our knowledge, this is the first report to investigate the detailed position of this plane. The position of the standard plane was at the plane obtained by rotating the coronal plane posteriorly by an average of 8.27°, and this rotational angle had a significant correlation with the acetabular anteversion. We suggest that applying the US probe by tilting slightly posteriorly from the coronal plane may be useful for reducing the difficulty of the Graf method and for minimizing measurement errors.
